# A novel MSC-based immune induction strategy for ABO-incompatible liver transplantation: a phase I/II randomized, open-label, controlled trial

**DOI:** 10.1186/s13287-021-02246-4

**Published:** 2021-04-16

**Authors:** Yingcai Zhang, Jiebin Zhang, Huimin Yi, Jun Zheng, Jianye Cai, Wenjie Chen, Tongyu Lu, Liang Chen, Cong Du, Jianrong Liu, Jia Yao, Hui Zhao, Guoying Wang, Binsheng Fu, Tong Zhang, Jian Zhang, Genshu Wang, Hua Li, Andy Peng Xiang, Guihua Chen, Shuhong Yi, Qi Zhang, Yang Yang

**Affiliations:** 1 Department of Hepatic Surgery and Liver Transplantation Center of the Third Affiliated Hospital, Organ Transplantation Institute, Sun Yat-sen University, Guangzhou, 510630 Guangdong China; 2 Organ Transplantation Research Center of Guangdong Province, Guangzhou, 510630 China; 3 Guangdong Key Laboratory of Liver Disease Research, Key Laboratory of Liver Disease Biotherapy and Translational Medicine of Guangdong Higher Education Institutes, the Third Affiliated Hospital of Sun Yat-sen University, Guangzhou, 510630 China; 4 Surgical Intensive Care Unit, The Third Affiliated Hospital of Sun Yat-sen University, Guangzhou, 510630 China; 5 Cell-gene Therapy Translational Medicine Research Center, The Third Affiliated Hospital of Sun Yat-sen University, Guangzhou, 510630 Guangdong China; 6 Center for Stem Cell Biology and Tissue Engineering, Key Laboratory for Stem Cells and Tissue Engineering, Ministry of Education, Sun Yat-Sen University, Guangzhou, 510080 China

**Keywords:** Mesenchymal stem cells, ABO-incompatible liver transplantation, Severe hepatic failure, Rituximab

## Abstract

**Background:**

ABO-incompatible liver transplantation (ABO-i LT) has become a rescue therapeutic option for patients with severe hepatic failure. Although the use of rituximab greatly reduces the morbidity of antibody-mediated rejection (AMR), severe adverse effects, such as infection and biliary complications, still seriously threaten the survival of transplant recipients. The aim of this study was to evaluate the safety and feasibility of using mesenchymal stem cells (MSCs) to replace rituximab in ABO-i LT.

**Methods:**

Twenty-two patients with severe hepatic failure undergoing ABO-i LT were enrolled and randomly divided into two groups: the MSC group and the rituximab group. The safety of the application of MSCs and the incidence of allograft rejection, including antibody-mediated rejection (AMR) and acute cellular rejection (ACR), were evaluated in both groups at the 2-year follow-up period as primary endpoints. Recipients and graft survival and other postoperative complications were compared as secondary endpoints.

**Results:**

No severe MSC-related adverse events were observed during the trial. MSC treatment yielded comparable, if not better, results than rituximab at decreasing the incidence of acute rejection (9.1% vs 27.3%). Inspiringly, compared to those in the rituximab group, the rates of biliary complications (0% vs 45.5%) and infection (9.1% vs 81.8%) were significantly decreased in the MSC group. In addition, there were no significant differences in 2-year graft and recipient survival between the two groups (81.8% vs 72.7%).

**Conclusions:**

Our data show that MSC transfusion is comparable to rituximab treatment for AMR prophylaxis following ABO-i LT. Additionally, the results indicate that MSCs are more beneficial to the prevention of infection and biliary complications and may be introduced as a novel immunosuppressive approach for ABO-i LT.

**Trial registration:**

Trial registration: chictr.org.cn, ChiCTR2000037732. Registered 31 August 2020- Retrospectively registered, http://www.chictr.org.cn/showproj.aspx?proj=57074.

## Background

Severe hepatic failure is a life-threatening illness with high mortality and morbidity that is pathologically characterized by sudden and severe hepatocellular necrosis and clinically characterized by coagulopathy, jaundice, and hepatic encephalopathy [1]. Emergency liver transplantation (LT) is still identified as the only durable effective therapeutic approach. Unfortunately, the implementation of ABO-compatible LT is limited and sometimes unavailable due to global donor shortages [2]. Therefore, ABO-incompatible (ABO-i) grafts have become an alternative option for LT. The first ABO-i LT was conducted and reported in 1972, and the technique tends to be reserved for patients at higher risks of hepatic artery thrombosis, bile duct complications, antibody-mediated rejection (AMR), infection and poor graft, and recipient survival. According to Guggenheim et al., graft function in the first 2 years after LT in the ABO-i group (30%) was significantly lower than that in the ABO-compatible group (76%) [3]. To overcome these disadvantages, various novel therapeutic strategies have been introduced over the past two decades, including an anti-CD20 monoclonal antibody (rituximab), intravenous immunoglobulin (IVIG), splenectomy, immunoadsorption, and plasma exchange. Similar prognostic outcomes and recipient survival rates of ABO-c LT were observed in the ABO-i LT population when rituximab was combined with desensitization [4], and further multivariate analysis demonstrated that the absence of rituximab administration was an independent risk factor for AMR [5]. However, a retrospective study with a large sample size also reported an incidence of bile duct injury after treatment with rituximab [6].

With the properties of immunomodulation and regeneration, mesenchymal stem cells (MSCs) are emerging as a promising approach for many diseases, including acute-on-chronic hepatic failure, rheumatoid arthritis, and inflammatory bowel disease. Due to their important roles in modulating the function of macrophages, natural killer (NK), T cells, and B cells and further inducing the translation of Treg and Breg cells, MSCs are believed to prevent postoperative complications after transplantation and reduce the side effects of pharmacologic immunosuppression [7, 8]. In a previous trial, Tan et al. showed that autologous MSC administration achieved a lower incidence of acute rejection, a lower risk of opportunistic infection, and better renal function restoration after kidney transplantation than anti-IL-2 receptor monoclonal antibody treatment [9]. Detry once reported the use of MSC treatment for liver transplant recipients and showed no toxic or severe side effects after a single-dose MSC transfusion [10]. Wang et al. showed that umbilical cord-derived MSC transfusion is feasible for inhibiting acute graft rejection after LT by increasing the percentage of Treg cells and the Treg/Th17 ratio [11]. Our previous study also revealed the beneficial effect of MSCs on attenuating ischemia-type biliary lesions (ITLBs) after LT [12]. However, clinical research on MSC administration in ABO-i LT has not been conducted.

To our knowledge, this was the first prospective, rituximab-controlled, clinical phase I/II study to focus on the safety, tolerability, and feasibility of intravenous transfusion of multidose allogeneic MSCs in severe hepatic failure patients who underwent ABO-i LT. Potential side effects of MSC administration and postoperative complications, especially acute rejection, biliary complications, and infection, over 2 years after the operation were investigated as the primary endpoints. The secondary endpoints were set to clinically compare the recipients and graft survival rates between the MSC and rituximab groups.

## Methods

### Study design and participants

**Fig. 1:**
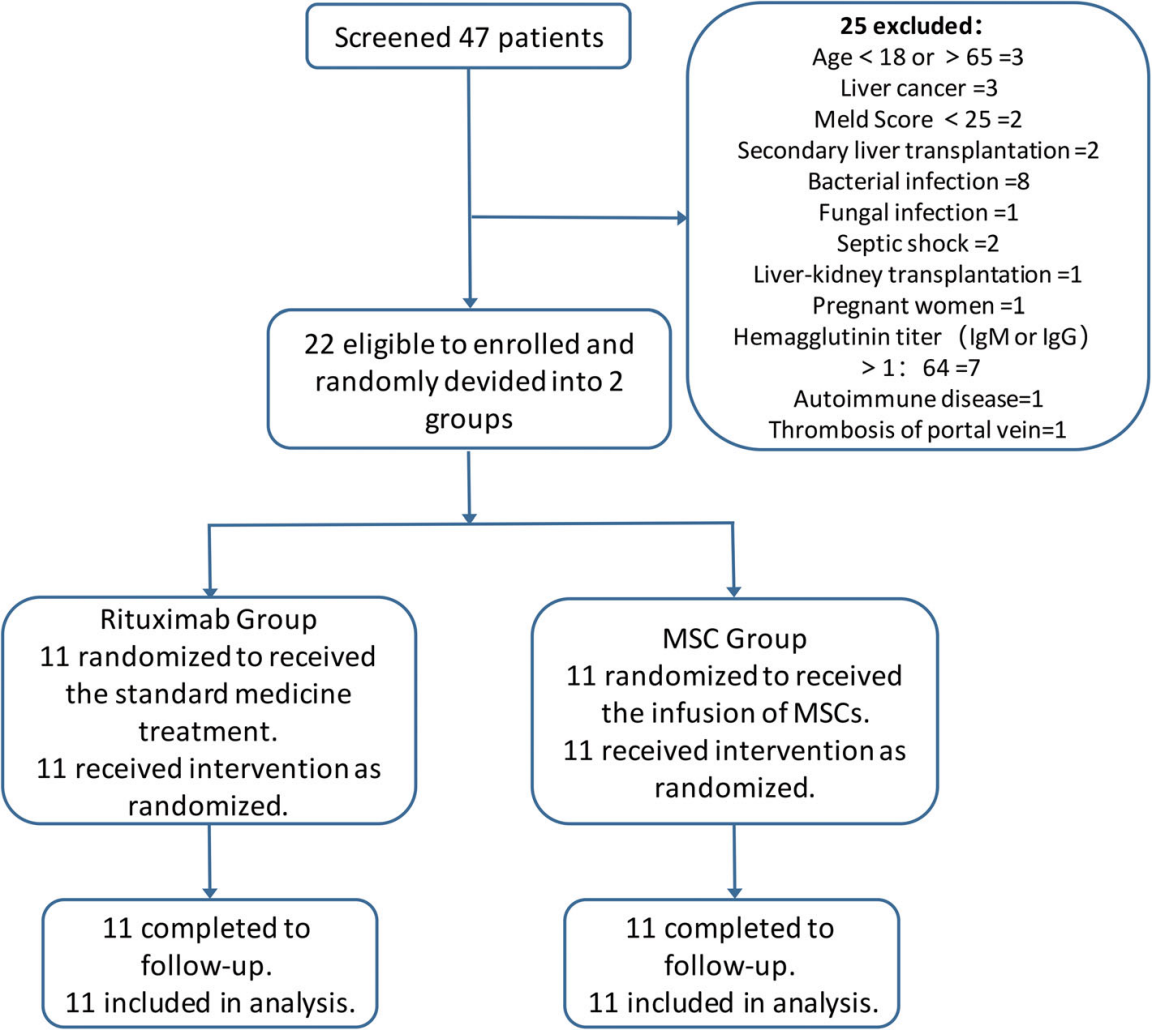
Study flow chart. Enrolment, randomization, and follow-up of patients in the MSCs and rituximab trial. Between August 2016 and August 2018, a total of 47 severe hepatic failure patients receiving ABO-i LT were screened. Twenty-two participants were enrolled in the present study and were randomly divided into 2 groups (MSC group = 11, rituximab group = 11). Finally, 11 participants in the MSC group and 11 in the rituximab group completed the follow-up and were included in the analysis

The current study was a prospective, monocentric, open-label, randomized, rituximab-controlled, phase I/II clinical trial that featured a total 2-year follow-up period. This trial was approved by the local ethics committee on clinical trials of the Third Affiliated Hospital of Sun Yat-sen University (Guangzhou, China), which piloted conforming to the ethics guideline of the 1975 Declaration of Helsinki and registered the trial at chictr.org.cn (ChiCTR2000037732). The target population involved adults with severe hepatic failure who were hospitalized for an emergency ABO-i LT. The detailed inclusion and exclusion criteria are listed in Fig. 1.

All participants were recruited at the Department of Liver Transplantation of the Third Affiliated Hospital of Sun Yat-sen University. Between August 2016 and August 2018, 22 patients were enrolled after screening a total of 47 patients receiving ABO-i LT at our center, and the enrolled patients were randomly assigned equally to the MSC or rituximab group. All patients in this trial provided written informed consent.

**Fig. 2:**
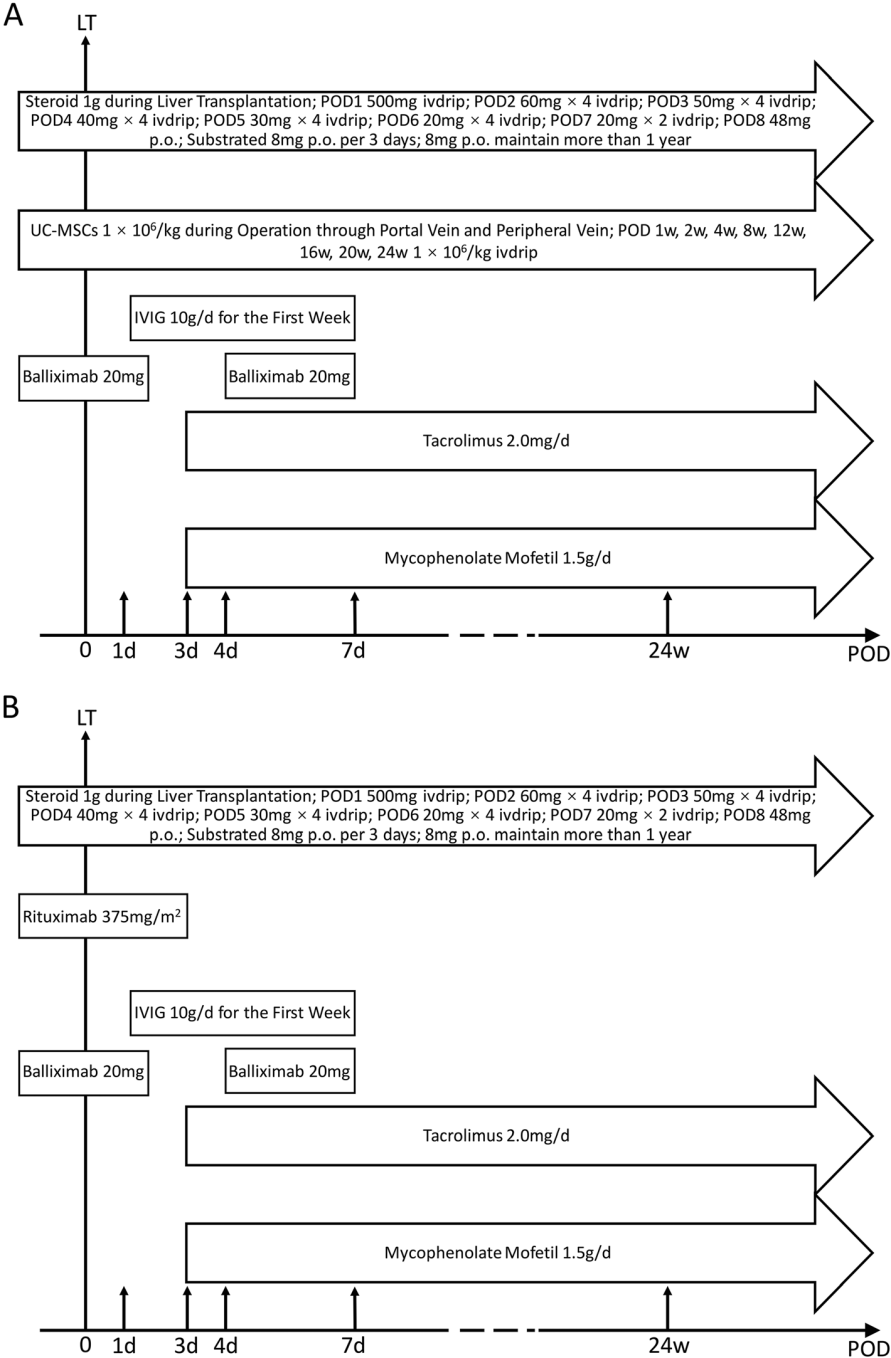
Immunosuppression protocol for ABO-i LT in the MSC (**a**) and rituximab trial (**b**). LT liver transplantation; IVIG intravenous immunoglobulin

All enrolled patients in these two groups received standard immunosuppressive regimen treatment according to previously described methods, including steroids, baliximab, tacrolimus, mycophenolate mofetil, and intravenous immunoglobulin (IVIG) [13]. Specifically, 1 g of steroid was administered during the operation, followed by 500 mg on day 1 and 240 mg on day 2. The dose was tapered by 40 mg/d until the daily dose reached 40 mg/d. Subsequently, the steroid was administered at 48 mg/d p.o., with the dose tapered by 8 mg every 3 days until reaching 8 mg/day, and this does was then maintained at 8 mg/d for more than 1 year. IVIG (10 g/d) was used in the first 7 days after LT. Tacrolimus (2.0 mg/d) and mycophenolate mofetil were initially administered on the third day after ABO-i LT to maintain the blood concentrations of the drugs. Finally, baliximab was given twice, once during surgery and again on the fourth day after transplantation (Fig. 2).

### Liver transplant procedures

The following details regarding the enrolled deceased liver graft donors were prospectively recorded: sex, age, donor after circulatory or brain death (DCD or DBD), cause of death, body mass index (BMI), ABO blood type, terminal serum sodium level, terminal hepatic and renal function tests, sojourn time in the intensive care unit (ICU), ventilator settings, and need for vasopressors.

The ABO-i LT procedures, Piggy-back LT, were standardly performed at the authors’ single centers, which were specified in detail previously [14]. The following recipient characteristics were collected: sex, age, liver, and renal function tests; coagulation function tests; model for end-stage liver disease (MELD) score; and titers of specific antibodies upon hospitalization for LT. During transplantation, the operation time, cold graft ischemic time, intraoperative blood loss, and blood transfusion volume were recorded.

### Preparation, culture, and identification of allogeneic MSCs

#### Umbilical cord donors

Human umbilical cords were obtained from healthy donors who understood the study, met the inclusion and exclusion criteria and provided written informed consent. Before preparing the MSCs, the umbilical cords were initially confirmed to be negative for cytomegalovirus (CMV) antigen, anti-human T lymphotrophic virus (HTLV) I/II antibody, anti-hepatitis A virus (HAV) IgM antibody, hepatitis B virus (HBV) antigen, anti-hepatitis C virus (HCV) antibody, hepatitis D virus (HDV) antigen, anti-hepatitis E virus (HEV) IgM/IgG antibodies, syphilis, anti-HIV-1/2 antibodies, fungi, and bacteria. In addition, we limited the age of the donors from 18 to 35.

#### Preparation, culture, and identification of MSCs

Third-party MSC preparation was approved by the Ethics Committee of the Third Affiliated Hospital of Sun Yat-sen University. Following good manufacturing practice (GMP), the cell preparation and culture processes were performed under standardized and aseptic conditions at the Stem Cell Laboratory Facility of the Biotherapy Center at our hospital [12]. Fresh UCs were obtained from healthy pregnant women and immersed in 4 °C phosphate-buffered saline (PBS). The umbilical cords were washed twice with PBS to remove the remnant blood until they became white, cut into 10 mm^3^/piece in 0.1% type I collagenase with CaCl_2_ (3 mM) containing 0.1% hyaluronidase (Invitrogen, USA), and then incubated on a shaker (220 rpm) at 37 °C for 4 h digestion. Subsequently, the isolated cells were cultured in low-sugar Dulbecco’s modified Eagle’s medium (1 g/L DMEM, Gibco, Life, Austria) with 10% fetal bovine serum (FBS, Gibco, Life, Austria) in a humidified atmosphere of 5% CO_2_ and 37 °C. The medium was refreshed every 3 days to remove nonadherent cells. To assess the phenotype on the cell surface, flow cytometric analysis of CD105, CD73, CD44, CD90, CD45, CD34, CD166, and CD29 was performed. The differential potential was detected according to criteria established by the 2006 International Society of Cellular Therapy, which investigated their ability to differentiate into osteocytes and adipocytes [15]. At 70–80% confluence, MSCs were passaged by trypsin treatment. MSCs were collected and clinically used at passages 3–5. Before injection, the cells were tested again and confirmed to be negative for HBV, HCV, HIV, syphilis, mycoplasma, fungi, and endotoxins.

#### Allogeneic MSC transfusion

Allogeneic MSCs were collected and suspended in 100 ml of 0.9% NaCl at a density of 1.0 × 10^6^ cells/kg body weight. The enrolled patients received nine total doses of MSC infusion, including the first time, which involved the infusion of 10% of the MSCs through the portal vein after graft reperfusion and 90% of the MSCs being transfused through the peripheral vein during LT; the subsequent 8 infusions (1 week, 2 weeks, 4 weeks, 8 weeks, 12 weeks, 16 weeks, 20 weeks, and 24 weeks after operation) were administered intravenously via the forearm. During the infusion, the MSC suspension was transfused within 30 min and swung gently every 3 min to avoid cell deposition. All patients were observed for 2 h after the transfusion to screen for any adverse events.

#### Primary and secondary endpoints

The primary endpoints of the investigation were the safety and tolerability of multidose MSC administration in study subjects with assessments of MSC-related adverse events (fever, headache, rash, vomiting, diarrhea, and carcinogenesis) and the incidence of allograft rejection, including antibody-mediated rejection (AMR) and acute cellular rejection (ACR), at the 2-year follow-up period. The secondary endpoints were the preliminary observations of MSC efficacy in patients who underwent ABO-i LT compared with rituximab, including (1) the evaluation of graft and recipient survival and (2) the incidence of postoperative complications, including biliary complications and specific infections.

#### Graft biopsy and immunohistochemistry

At week 2 and month 6, surveillance biopsies were performed, and 4-μm formalin-fixed, paraffin-embedded sections were prepared. First, hematoxylin and eosin (H&E) staining was performed to characterize graft rejection according to the Banff criteria by double-blind scoring [16]. In addition, the sections were stained with cytokeratin 19 (CK19) to observe biliary formation, stained with C4d to indirectly assess the severity of AMR, and immunostained with antibodies against human CD4, CD8, and CD20 to assess immune cell infiltration. At least three fields (×200 magnification) from each patient were randomly selected to calculate the mean number of positive cells. Archival tonsil sections were used as positive controls. Sections treated with only the secondary antibody and diaminobenzidine (no primary antibody) served as the negative controls. All of the primary antibodies were purchased from Abcam (USA), and the secondary antibody and diaminobenzidine were purchased from DAKO (USA).

#### Definitions

Acute cellular rejection (ACR) was scored using the Banff criteria. AMR was serologically diagnosed by acute tissue injuries, such as vascular inflammation and bile duct inflammation damage, and significantly increased titers of specific antibodies and histologically diagnosed by C4d staining [17]. Hepatic arterial stenosis was defined by visualizing Doppler ultrasonography and computed tomographic (CT) angiography. Biliary leakage was suspected with the persistent drainage of bile from the abdominal cavity and diagnosed by postoperative cholangiography. Bile duct anastomotic stenosis was diagnosed by magnetic resonance cholangiopancreatography (MRCP). ITBL was suspected based on laboratory examinations and elevated levels of serum ALP and γ-GGT and diagnosed by contrast-enhanced ultrasonography (CEUS) and MRCP [18, 19]. Post-transplant septic shock was diagnosed in patients who suffered from severe sepsis, which was determined by a positive culture of pathogenic forms of bacteria or fungi, with hyperlactatemia and obvious hemodynamic changes requiring vasopressor therapy.

### Statistical analysis

As appropriate, summary data with continuous variables are presented as descriptive statistics, including *n*, mean ± standard deviation (SD), median/interquartile, and maximum/minimum, whereas the categorical variables are summarized using frequency and percentages. A mixed model (repeated measures) was used to analyze the outcomes from multiple follow-up times to compare the efficacies between the two groups, whereas comparisons within the same group were performed using the model-estimated contrasts. If the outcomes were highly skewed, a Wilcoxon signed rank sum test was used to make comparisons of each intragroup follow-up effect by covariance adjustment of the baseline. In addition, categorical variables were analyzed by Fisher’s exact tests. All data from this study were analyzed by SPSS 23.0 software (SPSS Inc., Chicago, IL, USA) or GraphPad software version 7^.^0 (CA, USA), as appropriate. Two-sided *p* < 0.05 was considered statistically significant.

## Results

### Baseline characteristics of recipients and donors

The baseline characteristics of the recipients and donors are listed in Table 1. From August 2016 to August 2018, 22 severe hepatic failure patients were enrolled to receive ABO-i LT at the Third Hospital of Sun Yat-sen University. The patients were randomly divided into two groups: the rituximab group (*n* = 11) and the MSC group (*n* = 11) (Fig. 1). Up to August 2020, the median follow-up period was 32 months in the MSC group (4 days–48 months) and 37 months in the rituximab group (2–47 months). Among all the patients, the most common primary disease (20/22) was hepatitis B virus-related acute-on-chronic liver failure. The receipt age, preoperative WBC count, and CPS scores were comparable between the rituximab and MSC groups. There was no significant difference in the MELD scores between the MSC and rituximab groups (*p* = 0.217). The data of the blood type combinations between donors and recipients are provided with the isoagglutinin titer in Table 2. The most common combination of the ABO type of donor to recipient was AB to A (9/22). Both initial IgM and IgG isoagglutinin titers were ≤ 1:64. No significant differences in the isoagglutinin titer were detected in the pretransplantation period. In addition, the donor characteristics, especially the cold ischemic time, as well as the duration of transplant surgery and transfusions, were comparable between MSC- and rituximab-treated recipients.

**Table 1:** Clinical and biochemical index of the patients at baseline

	MSC (***N*** = 11)	Rituximab (***N*** = 11)	***P*** value
**Gender (male)(%)**	9 (81.8%)	10 (90.9%)	1.000
**Age (years)**	48.64 ± 7.94	43.64 ± 13.25	0.296
**WBC (× 10**^**9**^**)**	7.32 (3.86–11.60)	9.29 (6.69–15.66)	0.365
**PLT (×10**^**9**^**/L)**	71.00 (47.00–90.00)	104.00 (75.00–201.00)	0.056
**AST (U/L)**	69.00 (21.00–111.00)	97.00 (53.00–180.00)	0.217
**ALT (U/L)**	30.00 (9.00–94.00)	99.00 (18.00–233.00)	0.171
**ALB (g/L)**	32.20 (30.80–35.80)	34.20 (33.20–36.20)	0.773
**TBIL (umol/L)**	420.73 (38.45–541.73)	590.07 (202.35–702.68)	0.116
**ALP (U/L)**	99.00 (95.00–124.00)	99.00 (71.00–116.00)	0.478
**GGT (U/L)**	49.00 (33.00–77.00)	51.00 (42.00–75.00)	1.000
**PT (s)**	25.60 (20.60–37.60)	33.20 (29.10–38.80)	0.251
**INR**	2.31 (1.75–3.67)	3.17 (2.71–3.98)	0.270
**CREAT (umol/L)**	76.00 (45.00–114.00)	71.00 (56.00–174.00)	0.748
**BUN (mmol/L)**	7.73 (5.01–9.16)	5.35 (3.18–15.44)	0.699
**IgM**	8.00 (8.00–32.00)	8.00 (8.00–16.00)	0.898
**IgG**	16.00 (8.00–32.00)	32.00 (2.00–64.00)	0.478
C**PS score**	11.82 ± 1.66	11.46 ± 1.57	0.604
**MELD** s**core**	31.00 (30.00–40.00)	40.00 (32.00–40.00)	0.217
**Donor age (years)**	41.09 ± 15.78	42.73 ± 8.63	0.767
**Cold ischemia time(h)**	6.00 (6.00–7.50)	6.00 (4.72–7.00)	0.270
**Operation time (min)**	443.64 ± 41.65	425.64 ± 60.23	0.425
**Anhepatic phase time (min)**	45.00 (42.00–55.00)	43^.^00 (40^.^00–55^.^00)	0^.^847
**Intraoperative blood loss (mL)**	1500.00 (1500.00–2400.00)	1500^.^00 (1000^.^00–2000^.^00)	0^.^365
**Blood transfusion volume**			
**RBC (U)**	12.00 (6.00–16.00)	9.50 (7.50–16.00)	0^.^797
**FFP (mL)**	2400.00 (2100.00–3600.00)	3050.00 (2000.00–4000.00)	0^.^606
**Cryo (U)**	28.00 ± 15.28	36.05 ± 8.08	0^.^138

Abbreviations: *MSC* mesenchymal stem cell, *WBC* white blood cell, *PLT* platelet, *AST* aspartate aminotransferase, *ALT* alanine aminotransferase, *ALB* albumin, *TBIL* total bilirubin, *ALP* alkaline phosphatase, *GGT* gamma-glutamyl transferase, *PT* prothrombin time, *INR* international normalized ratio, *CREAT* creatinine, *BUN* blood urea nitrogen, *CPS* Child-Pugh score, *MELD* model for end stage liver disease, *RBC* red blood cell, *FFP* fresh frozen plasma, *Cryo* cryoprecipitate

**Table 2:** Blood type combinations between donor and recipient with the isoagglutinin titer

Patient number	Donor blood type	Recipient blood type	Isoagglutinin titer IgM	Isoagglutinin titer IgG
**1**	AB	A	1:8	1:32
**2**	AB	A	1:64	1:2
**3**	A	O	1:32	1:64
**4**	B	O	1:8	1:32
**5**	AB	A	1:8	1:8
**6**	AB	A	1:32	1:16
**7**	AB	A	1:4	1:16
**8**	AB	A	1:8	1:8
**9**	B	A	1:8	1:4
**10**	A	B	1:4	1:8
**11**	A	O	1:8	1:64
**12**	B	O	1:16	1:64
**13**	AB	A	1:8	1:2
**14**	B	O	1:8	1:2
**15**	AB	O	1:32	1:32
**16**	B	O	1:16	1:64
**17**	B	O	1:8	1:64
**18**	AB	B	1:4	1:2
**19**	AB	A	1:8	1:16
**20**	AB	A	1:8	1:32
**21**	B	O	1:64	1:64
**22**	A	B	1:4	1:32

### Primary outcomes

#### Safety of MSC infusion in ABO-I liver transplant recipients

MSC infusions were performed according to the design scheme shown in Fig. 2. Most of the observed adverse events were of grades I/II and transient, including fever, headache, rash, vomiting, and diarrhea, which appeared to be closely associated with the administration of MSCs (Table 3). No significant variations in vital parameters (such as blood pressure, heart rate and SpO_2_) were detected during or after the MSC infusion. Moreover, no patients in the MSC group developed de novo cancerous complications (including post-transplant lymphoproliferative disease) during the 2-year follow-up period.

**Table 3:** Side effects after MSC infusion

	No.								
Adverse event	Time 2 (***n*** = 3)	Time 3 (***n*** = 4)	Time 4 (***n*** = 0)	Time 5 (***n*** = 2)	Time 6 (***n*** = 2)	Time 7 (***n*** = 3)	Time 8 (***n*** = 5)	Time 9 (***n*** = 3)	Total (***n*** = 25)
**Fever**	2	2	0	1	1	2	3	1	13
**Headache**	0	1	0	1	0	0	0	1	3
**Rash**	0	0	0	0	1	0	1	0	2
**Vomiting**	1	0	0	0	0	1	0	0	4
**Diarrhea**	0	1	0	0	0	0	1	1	3

#### Incidence of AMR and ACR

**Fig. 3:**
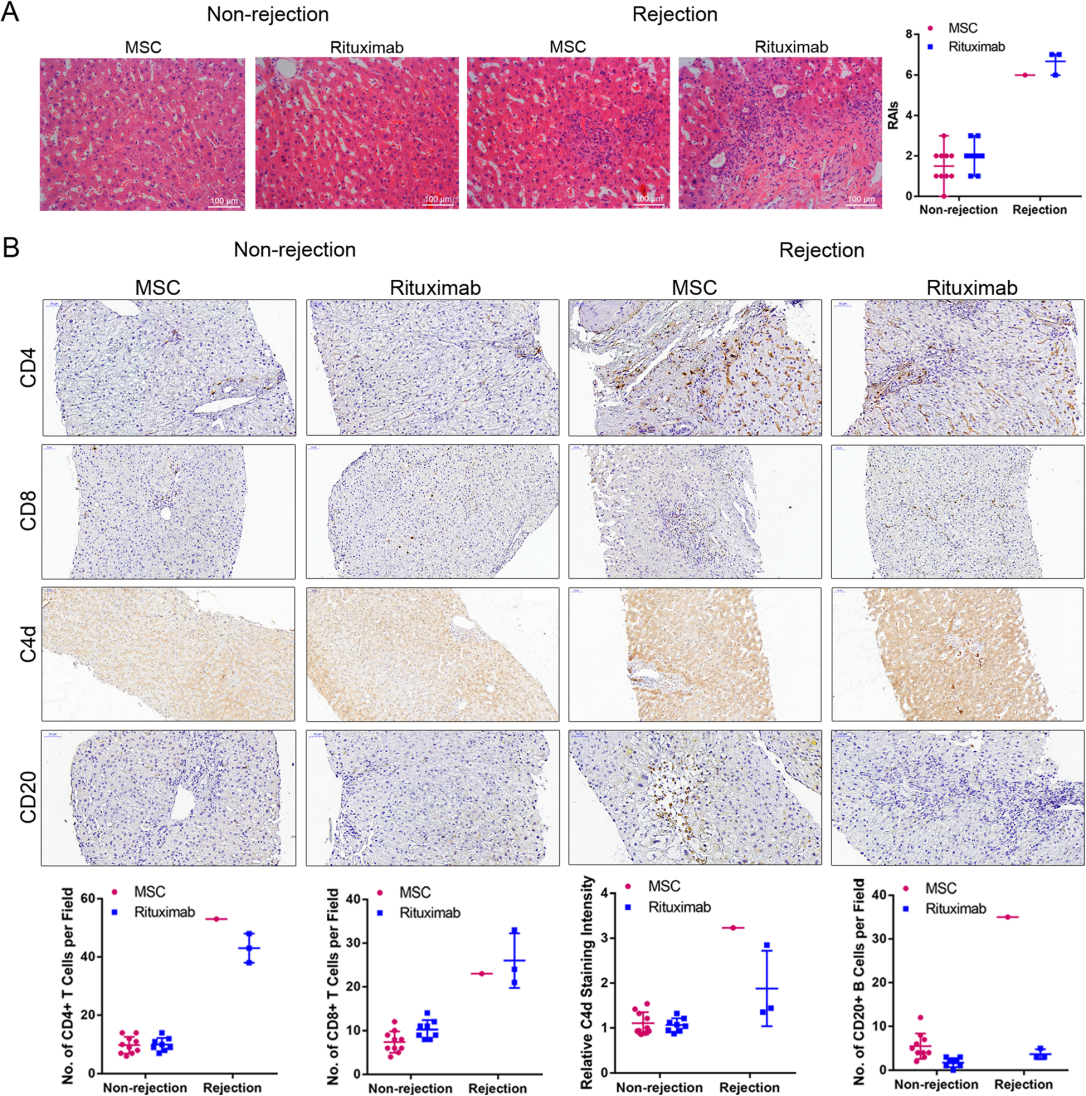
Sixth-month graft biopsies. **a** Representative sections of livers stained with hematoxylin and eosin (H&E), including cases of nonrejection and rejection during MSC or rituximab treatment (×200 magnification). Severe portal vein endotheliitis and bile duct damage in liver biopsy specimens were observed with rejection (RAIS: 6–7). Mild portal inflammation and bile duct inflammation and damage were observed without rejection (RAIS: 1–3). RAIS=P(n1) + V(n2) + B(n3). **b** Representative IHC images of CD4, CD8, C4d, and CD20 staining, including cases of nonrejection and rejection during MSC or rituximab treatment (× 200 magnification). The positive staining of CD4 and CD8 reflects the inflammatory infiltration of T cells with rejection. The positive staining of C4d and CD20 suggests AMR. Abbreviations: MSC mesenchymal stem cell, RAIS rejection activity indexes, P(n1) scores for portal inflammation, V(n2) scores for venous endothelial inflammation, B(n3) scores for bile duct inflammation damage, AMR antibody-mediated rejection

Several studies have demonstrated that the incidences of antibody-mediated rejection (AMR) and acute cellular rejection (ACR) are much more frequent in ABO-i recipients than in ABO-compatible recipients. As illustrated in Table 4, three patients in the rituximab group developed acute liver rejection reactions at days 20, 43, and 78, while only one patient in the MSC group developed acute liver rejection reactions at day 25. MSC therapy significantly decreased the rate of acute rejection compared with the rituximab group in the follow-up period (9.1% vs. 27.3%, *p* = 0.586). Hematoxylin and eosin (H&E) staining of liver tissue sections reflected the rejection or nonrejection through acute tissue injury, including vascular inflammation and bile duct inflammation damage (Fig. 3a). Immunohistochemistry of CD4 and CD8 demonstrated that T lymphocytes strikingly infiltrated the hepatic tissues in patients with acute allograft rejection. Moreover, C4d staining showed one instance of AMR in each group (Fig. 3b). The symptoms were relieved by increasing the dosage of the current immunosuppressive agents.

**Table 4:** Prognosis in 2 years after liver transplantation

	MSC (***N*** = 11)	Rituximab (***N*** = 11)	***P*** value
**Survival rate no. (%)**	9 (81.8%)	8 (72.7%)	1.000
**Graft survival rate no. (%)**	9 (81.8%)	8 (72.7%)	1.000
**Acute rejection no. (%)**	1 (9.1%)	3 (27.3%)	0.586
**Biliary complications no. (%)**	0 (0%)	5 (45.5%)	0.035^a^
**Ischemia type biliary lesion no. (%)**	0 (0%)	4 (36.4%)	0.090
**Bile duct anastomotic stenosis no. (%)**	0 (0%)	3 (27.3%)	0.214
**Bile leakage no. (%)**	0 (0%)	1 (9.1%)	1.000
**Septic shock no. (%)**	1 (9.1%)	9 (81.8%)	0.002^b^
**Pulmonary infection no. (%)**	0 (0%)	8 (72.7%)	0.001^b^
**Biliary infection no. (%)**	0 (0%)	1 (9.1%)	1.000
**Splenic abscess no. (%)**	1 (9.1%)	0 (0%)	1.000
**Arteriostenosis no. (%)**	1 (8.3%)	3 (27.3%)	0.586
**De novo tumor no. (%)**	0 (0%)	1 (9.1%)	1.000

^a^*P* < 0.05, ^b^*P* < 0.01

Abbreviations: *MSC* mesenchymal stem cell

### Secondary outcomes

#### Effects of MSCs on patient and graft survival

Five recipients died during the follow-up period. In detail, two patients in the MSC group died of abdominal hemorrhage (*n* = 1) and hepatic failure caused by compressed inferior vena cava (*n* = 1). Three patients in the rituximab group died of sepsis-associated multiple organ failure, including two patients who died due to severe pulmonary (*n* = 1) and biliary tract infection (*n* = 1) within 3 months after transplantation and one patient who died in the sixteenth month from pulmonary infection-induced septic shock and refractory anemia. The 2-year graft and recipient survival rates were 81.8% (9/11) and 72.7% (8/11) in the MSC and rituximab groups, respectively.

#### Effects of MSCs on liver graft function

**Fig. 4:**
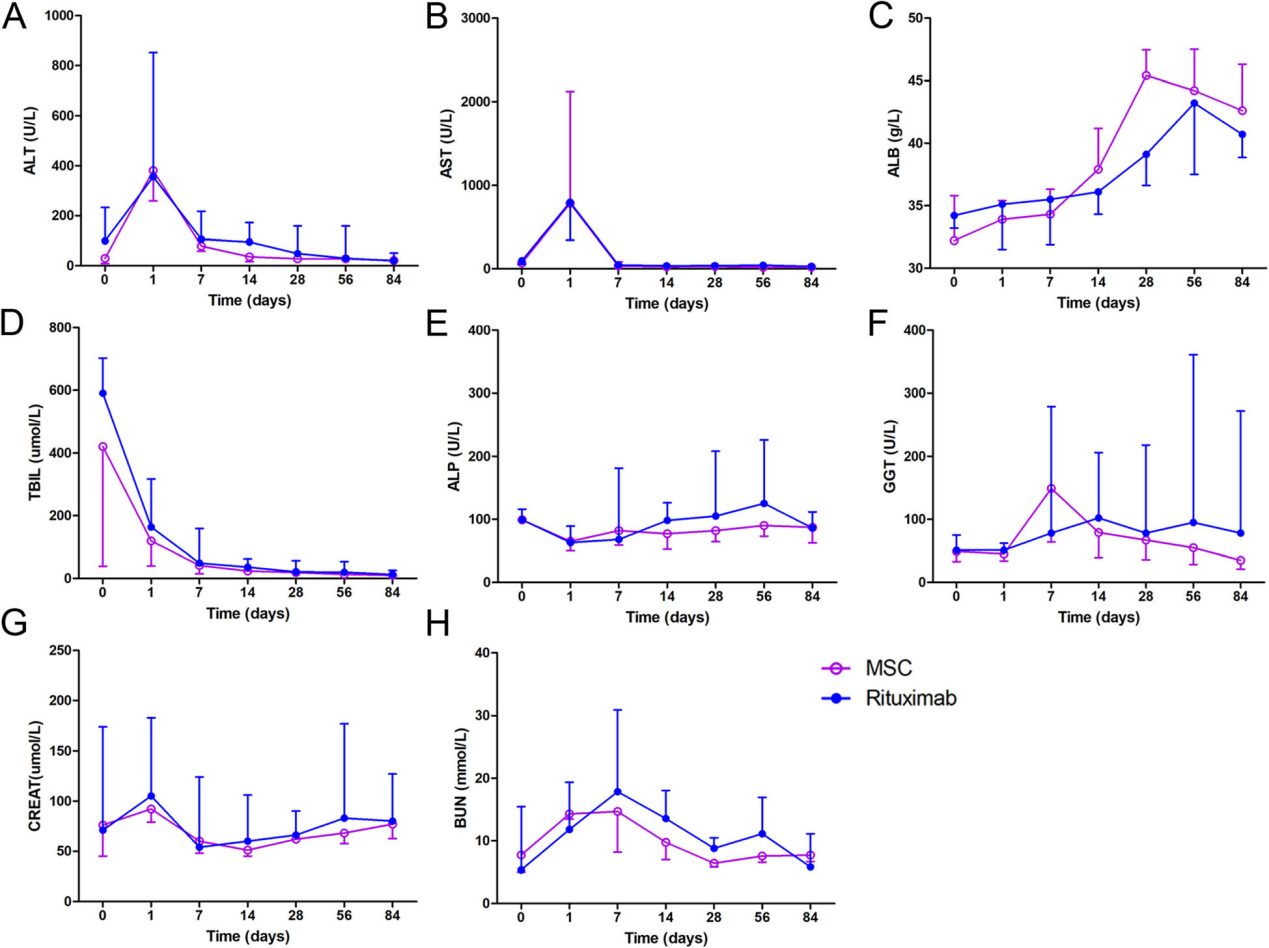
Postoperative laboratory tests. Including **a** ALT (U/L), **b** AST (U/L), **c** ALB (g/L), **d** TBIL (μmol/L), **e** ALP (U/L), **f** γ-GGT (U/L), **g** CREAT (μmol/L), and **h** BUN (mmol/L). (The data are presented as the mean ± SEM; *p < 0.05)

As shown in Fig. 4 and Table 5, to investigate the impact of MSCs on liver function, we measured the alanine aminotransferase (ALT), aspartate aminotransferase (AST), albumin (ALB), total bilirubin (TBIL), alkaline phosphatase (ALP), gamma glutamyl transferase (GGT), serum creatinine (CREAT), and blood urea nitrogen (BUN) levels in all patients throughout the follow-up period. Both ALT and AST reached their peak on the first day and then decreased to nearly normal levels. No significant differences were detected at any time point in either group. ALB showed an upward trend after MSC infusion, and the levels were especially higher than those in the rituximab group at week 4; however, there were no significant differences between the MSC and rituximab groups. TBIL levels were decreased significantly after MSC infusions compared with those in the rituximab group mainly within the first week. Although the ALP, GGT, CREAT, and BUN levels were not significantly different between the two groups, the fluctuation range in the MSC group was much milder than that in the rituximab group.

**Table 5:** Levels of AST, ALT, ALB, TBIL, ALP, GGT, CREAT, and BUN in the two groups at baseline, day 1, and weeks 1, 2, 4, 8, and 12

	AST			ALT			ALB			TBIL		
	Rituximab	MSC	***P***	Rituximab	MSC	***P***	Rituximab	MSC	***P***	Rituximab	MSC	***P***
**Baseline**	97.00 (53.00–180.00)	69.00 (21.00–111.00)	0.217	99 (18.00–233.00)	30 (9.00–94.00)	0.171	34.20 (33.20–36.20)	32.20 (30.80–35.80)	0.773	590.07 (202.35–702.68)	420.73 (38.45–541.73)	0.116
**Day 1**	794.00 (342.00–1032.00)	782.00 (308.00–2120.00)	0.748	355.00 (148.00–852.00)	380.00 (259.00–935.00)	0.652	35.10 (31.50–37.00)	33.90 (30.90–35.40)	0.411	163.07 (92.93–316.96)	119.00 (39.70–167.06)	0.193
**Week1**	45.00 (20.00–88.00)	34.00 (24.00–34.00)	0.898	106.00 (24.00–217.00)	79.00 (58.00–219.00)	0.652	35.50 (31.90–40.40)	34.30 (31.10–36.30)	0.263	48.69 (39.30–158.60)	41.64 (15.07–86.92)	0.365
**Week2**	33.00 (15.25–60.25)	18.50 (12.50–37.25)	0.387	67.50 (19.50–176.25)	36.00 (17.50–116.00)	0.426	36.10 (33.40–38.70)	36.90 (34.73–40.48)	0.644	42.05 (23.73–81.29)	19.87 (15.04–42.11)	0.314
**Week4**	38.00 (21.00–124.00)	28.00 (17.00–36.00)	0.261	49.00 (26.00–158.00)	28.00 (17.50–55.50)	0.295	39.10 (36.60–42.60)	45.40 (40.90–47.45)	0.01^*^	20.87 (17.14–56.74)	18.87 (11.00–28.58)	0.412
**Week8**	43.00 (19.00–67.00)	18.00 (14.50–39.00)	0.112	30.00 (16.00–158.00)	28.00 (15.50–91.50)	0.766	43.20 (37.50–44.50)	44.20 (41.60–47.50)	0.152	20.07 (12.70–53.24)	12.80 (8.90–15.51)	0.112
**Week12**	28.00 (20.00–32.00)	21.00 (18.00–40.00)	0.489	20.00 (13.00–50.50)	21.00 (13.50–96.50)	0.73	40.70 (38.85–45.65)	42.60 (41.85–46.30)	0.245	12.48 (8.42–25.97)	10.00 (9.45–12.40)	0.387
**Baseline**	99.00 (71.00–116.00)	99.00 (95.00–124.00)	0.478	51.00 (42.00–75.00)	49.00 (33.00–77.00)	1	71.00 (56.00–174.00)	76.00 (45.00–114.00)	0.748	5.35 (3.18–15.44)	7.73 (5.01–9.16)	0.699
**Day1**	63.00 (45.00–89.00)	65.00 (50.00–73.00)	0.478	51.00 (22.00–62.00)	45.00 (34.00–51.00)	1	105.00 (72.00–183.00)	92.00 (79.00–201.00)	1	11.79 (9.36–19.33)	14.28 (13.45–16.18)	0.519
**Week1**	68.00 (59.00–181.00)	82.00 (59.00–103.00)	0.949	78.00 (39.00–279.00)	149.00 (64.00–203.00)	0.949	54.00 (44.00–124.00)	60.00 (48.00–84.00)	0.898	17.84 (11.15–30.88)	14.68 (8.19–17.85)	0.27
**Week2**	99.50 (70.50–135.75)	67.00 (47.50–95.50)	0.24	103.50 (76.00–215.25)	61.50 (35.75–139.00)	0.705	68.50 (48.50–112.50)	49.50 (45.00–56.25)	0.282	14.48 (9.56–21.76)	10.97 (7.87–17.21)	0.152
**Week4**	105.00 (75.00–208.00)	82.00 (64.50–97.50)	0.112	78.00 (30.00–218.00)	67.00 (36.00–132.00)	0.656	66.00 (47.00–90.00)	62.00 (58.50–73.00)	0.656	8.78 (6.67–10.48)	6.40 (5.81–9.89)	0.503
**Week8**	125.00 (102.00–226.00)	90.00 (73.00–117.00)	0.056	95.00 (50.00–361.00)	55.00 (28.50–60.00)	0.031^*^	83.00 (56.00–177.00)	68.00 (57.50–95.00)	0.261	11.10 (4.32–16.94)	7.56 (6.56–10.06)	0.503
**Week12**	86.00 (69.00–111.50)	87.00 (62.50–130.50)	1	78.00 (42.00–272.00)	35.00 (21.00–92.50)	0.094	80.00 (73.50–127.00)	77.00 (62.50–95.00)	0.436	5.80 (5.21–11.12)	7.70 (6.68–8.32)	0.077

^*^P < 0.05

Abbreviations: *AST* aspartate aminotransferase, *ALT* alanine aminotransferase, *ALB* albumin, *TBIL* total bilirubin, *ALP* alkaline phosphatase, *GGT* gamma-glutamyl transferase, *CREAT* creatinine, *BUN* blood urea nitrogen, *MSC* mesenchymal stem cell

#### Post-transplant complications: infection

Due to the immunosuppressive effect of rituximab, opportunistic and severe infection is another common complication after ABO-i LT and might progress to septic shock and even multiple-organ failure. Thus, we investigated the impact of MSCs on the incidence of infection during the 2-year follow-up period (Table 4). Nine patients (9/11) developed pulmonary (*n* = 8) and biliary tract (*n* = 1) infection, two of whom died of severe and refractory pulmonary infection within 3 months after transplantation. On the other hand, only one patient (1/11) was diagnosed with a splenic abscess in the MSC group, indicating that the MSC treatment significantly decreased the rate of septic shock compared with the rituximab group (9.1% vs. 81.8%, *p* = 0.002).

#### Post-transplant complications: biliary tract complications

**Fig. 5:**
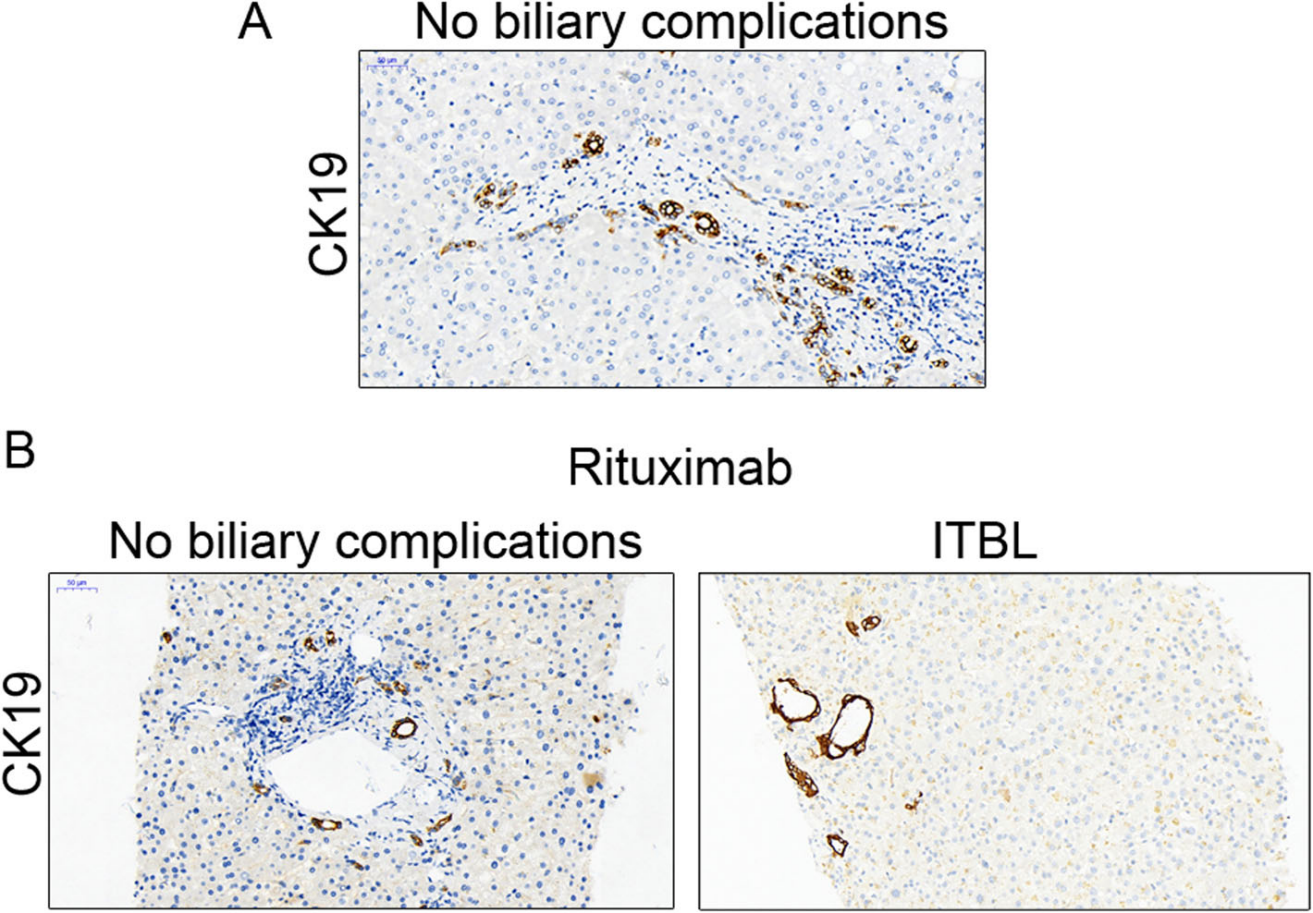
Sixth-month graft biopsies. Representative sections of the livers stained with CK19, including cases of no biliary complications and cases of ITBL during MSCs or rituximab treatment (× 200 magnification). **a** No biliary complications occurred in the MSC group, and the bile ducts stained with CK19 were normal. **b** CK19 staining of patients with or without biliary complications in the rituximab group. Abnormal changes included dilation and atresia of the bile ducts with ITBL

Biliary tract complications, including ischemic-type biliary lesions (ITBLs), biliary constriction, and fistula, are the major complications after ABO-i LT in patients using rituximab. In our study, the overall rate of biliary tract complications in the MSC group was significantly lower than that in the rituximab group (0% vs. 45.5%, *p* = 0.035) (Table 4). Our previous research proved the therapeutic effect of MSCs on biliary tract complications, especially ITBL. In this study, four patients in the rituximab group developed ITBL within 2 months after LT, and the incidence rate was much higher than that in the MSC group (36.4% vs. 0%, *p* = 0.09) (Table 4). In addition to magnetic resonance cholangiopancreatography (MRCP) and contrast-enhanced ultrasonography (CEUS), the diagnosis of ITBL was also confirmed by immunohistochemistry analysis of CK19 (Fig. 5). Adjustment of immunosuppressive agents with medication (*n* = 3) and interventional therapy (*n* = 1) were used to treat ITBL. In addition, biliary fistula was found in only one patient in the rituximab group and progressed to septic shock.

#### Post-transplant complications: others

In addition, as shown in Table 4, the arterial complications were comparable between the two groups (8.3% vs. 27.3%, *p* = 0.586). To our surprise, one patient in the rituximab group developed hepatocellular carcinoma, which was confirmed by pathological diagnosis. Thereafter, this patient received TACE treatment and is still alive now.

## Discussion

To our knowledge, we are the first to conduct a randomized, open-label clinical trial to evaluate the safety of the intravenous transfusion of allogeneic MSCs and compare the preliminary outcomes of MSCs and rituximab in recipients with severe hepatic failure receiving emergent ABO-i LT. Overall, this phase I/II study showed that multidose MSC administration was safe and well tolerated in this population. Furthermore, we also showed that as an adjunct treatment, transfusion of MSCs not only has a comparable ability to rituximab to prevent AMR but also has lower incidences of postoperative severe infections and biliary complications after ABO-i LT.

With the incredible capabilities of immunomodulation and organ protection, MSCs have been gradually praised as a promising therapy, and clinical trials on the administration of MSCs for various diseases have increased and shown encouraging outcomes. However, several issues still need to be discussed, and the source of MSCs is the first worth considering. Olivier et al. preferred the transfusion of BM-MSCs for patients after LT [10]. In addition, Wang et al. demonstrated the role of MSCs in attenuating acute graft rejection after LT by affecting the percentage of Tregs and the Treg/Th17 ratio [11]. In this study, we also used MSCs derived from the umbilical cords for patients after ABO-i LT, as the umbilical cords are more available and absolved of ethical problems and UC-MSCs are more capable of proliferation and immunomodulation than BM-MSCs [20–22]. At our center, MSCs were harvested at the Stem Cell Laboratory Facility of the Biotherapy Center at the Third Affiliated Hospital of Sun Yat-sen University and in accordance with standardized, aseptic requirements. Second, the doses and times of MSC administration are also controversial. The minimum effective cell dosage was suggested to be 1 × 10^7^ cells/kg, and the mortality rate was high when a single dose exceeded 21 × 10^7^/kg [23, 24]. However, due to the combined utilization of multiple immunosuppressants, we adopted 1 × 10^6^ cells/kg of single-dose MSC infusion in this study, which was similar to the suggestion in a previous study on MSC administration in organ transplantation fields [10, 25]. In addition, both single-dose and multiple-dose administrations were reported in past studies. Our previous clinical study showed the hepatoprotective effects of repeated doses of MSC transfusion for improving ITBL after LT without any severe side effects [12]. Thus, we continued to adopt this strategy in the current study and used eight doses of MSCs in the postoperative period. Furthermore, as a hyperacute rejection reaction theoretically occurs immediately after graft reperfusion, we added a dose in this period during the operation, including 10% of the MSCs administered through the portal vein after graft reperfusion and 90% of the MSCs being transfused through the peripheral vein [26].

As this is the first report on the administration of a stem cell-based therapy for preventing postoperative complications after ABO-i LT, safety issues remained the primary concern for our observations. Due to their characterization, MSCs are liable to embolize pulmonary circulation in animal experiments when they are transfused through peripheral or central veins to increase the burden of pulmonary exchange [27]. In addition, with the abilities of immunosuppression and multilineage differentiation, MSCs theoretically have a potential risk of carcinogenesis [28]. Fortunately, the results from this trial showed that our eleven recipients receiving MSC therapy did not develop severe infusional toxicity, including allergic reactions, or develop any signs of pulmonary dysfunction or malignant transformation after the 2-year follow-up. Only a few patients were noted to have a limited fever and to completely recover 3 h after the infusions without any special treatment. In general, our data indicated that allogeneic MSC administration for these patients was safe, which is consistent with our previous study on treating patients with ITBL after LT [12].

As another primary outcome, we additionally compared the incidence of allograft rejection between the rituximab and MSC groups. Traditionally, AR is divided into AMR and ACR. During ABO-i LT, a high ABO antibody titer may lead to a high risk of AMR. Rituximab is an immune chimeric monoclonal antibody that specifically targets the transmembrane protein CD20 molecule, which is expressed on the majority of B cells but not on antibody-producing plasma cells, to deplete B cells. Rituximab is also approved for application in the transplantation field, especially in ABO-i organ transplantation. In 2003, Monteiro et al. first reported the administration of rituximab for recipients receiving ABO-i LT, and since then, several studies have demonstrated that rituximab obviously reduces graft loss rates and is crucial to prevent the risk of AMR after ABO-i LT [29, 30]. In this setting, we found that MSC treatment resulted in a lower risk of AMR that was comparable to that of rituximab following ABO-i LT. This encouraging result also indicated that MSCs might replace rituximab to modulate the functions of B cells.

As the secondary outcome, we prospectively assessed the therapeutic effects of MSCs on ABO-i LT recipients by comparison with the rituximab group. No differences in the levels of ALT, ALP, AST, γ-GGT, BUN, CREAT, and TBIL were detected between the rituximab and MSC treatment groups during the follow-up period. Interestingly, of these results, we found a more distinct rise in the post-transplant ALB levels in the MSC group than in the rituximab group, which might suggest that MSCs play an important role in repairing liver function [31]. In addition, we also compared the rates of opportunistic infections between these two groups because both are immunosuppressive. A previous study showed that along with the effect on preventing AMR, rituximab increased the incidence of severe infections [32]. Again, a large-sample trial of multiple sclerosis showed that the rate of serious infections after rituximab use was higher than that in the control group [33]. Another study showed that MSC treatment for living-related kidney transplant recipients resulted in a lower rate of infectious complications [9]. In the present study, a higher rate of serious infection was observed in the rituximab group than in the MSC group. Three patients died from infection after treatment with rituximab, and this did not occur in the MSC group. Through liver biopsy, we also determined that compared with those in MSC-treated patients, the CD20-positive B cells in the liver tissues of patients treated with rituximab were obviously depleted, which may explain why patients treated with rituximab are susceptible to opportunistic infection. Other indicators, including the survival rate, graft survival rate, acute rejection, and arteriostenosis, were also not significantly different between these two groups with the exception of biliary complications. A large-size retrospective study by Song et al. demonstrated that biliary complications were the only concern regarding ABO-i LT after treatment with rituximab [6]. A likely high rate of ITBL in the rituximab group was observed in this study. Consistent with our previous studies, MSCs also exhibited the ability to protect biliary structure following ABO-i LT [12]. To explain this issue, we referred to past studies and found that after ABO-i LT kidney transplantation, the graft vascular endothelium markedly expressed ABO blood group antigens that were the target of attack [34]. Lacob et al. revealed that the presence of anti-human leucocyte antigen (HLA) class II antibodies was closely associated with biliary injury after LT [35]. We speculated that MSCs might play critical roles in regulating the level of DSA and affecting the membranous expression of MHC-II in the bile duct epithelium following ABO-i LT to improve biliary injury.

There are several important shortcomings in this study that should be acknowledged for improving further investigation. First, this phase I/II trial is the first study to enroll only 11 ABO-i LT recipients in both the MSC group and the rituximab group. Therefore, a well-designed study with a large sample size and a multicenter, a long-term investigation is required to further ascertain these outcomes. Second, due to the characteristics of ABO-i LT complications, we administered repetitive infusions of MSCs both during and after the operation via the portal and peripheral veins. However, the timing, dose, and route of MSC administration deserve deliberation and should be further evaluated. Furthermore, this was an open-label study, and neither the recipients nor observers were blinded to the therapeutic strategy. Thus, bias was inevitable in the interpretation of adverse events. Moreover, due to the lack of available tracer methods for clinical administration, we still did not clarify the traces or fates of MSCs after transfusion in these recipients. Finally, although we detected changes in the infiltration of several immune cells in the liver tissues in these two groups by immunohistochemistry staining during the follow-up period, the mechanism underlying the effect of MSCs in ABO-i LT should be further observed by evaluating the alterations of cytokines and immune cell subpopulations in the peripheral circulation.

## Conclusions

The present study is the first prospective, controlled clinical study evaluating the feasibility and safety of allogeneic MSC transfusion in a series of patients with severe hepatic failure receiving ABO-i LT. In this study, no severe side effects of MSC transfusion after surgery were found. In addition to AMR prevention, MSC administration effectively reduced the incidence of opportunistic infection and biliary complications compared with rituximab. Although this is a small cohort study, the knowledge that we have gained regarding the biological effect of MSCs in ABO-i LT allowed us to conduct a large and randomized study to further confirm the feasibility of MSC treatment for preventing postoperative complications after ABO-i LT (Chictr.org.cn: ChiCTR2000037732).

## Data Availability

The datasets used and analyzed during the current study are available from the corresponding author upon reasonable request.

## Abbreviations

ABO-i LT: ABO-incompatible liver transplantation
SHF: Severe hepatic failure
AMR: Antibody-mediated rejection
MSC: Mesenchymal stem cell
IVIG: Intravenous immunoglobulin
IBD: Inflammatory bowel disease
NK: Natural killer
ITLBs: Ischemia-type biliary lesions
MMF: Mycophenolate mofetil
DCD: Donor after circulatory death
DBD: Donor after brain death
ICU: Intensive care unit
MELD: Model for end-stage liver disease
CMV: Cytomegalovirus
HTLV: Human T lymphotrophic virus
HAV: Hepatitis A virus
HBV: Hepatitis B virus
HCV: Hepatitis C virus
HDV: Hepatitis D virus
HEV: Hepatitis E virus
GMP: Good manufacturing practice
PBS: Phosphate-buffered saline
FBS: Fetal bovine serum
AR: Allograft rejection
ACR: Acute cellular rejection
H&E: Hematoxylin and eosin
CK19: Cytokeratin 19
CT: Computed tomographic
MRCP: Magnetic resonance cholangiopancreatography
CEUS: Contrast-enhanced ultrasonography
HLA: Human leucocyte antigen
WBC: White blood cell
PLT: Platelet
AST: Aspartate aminotransferase
ALT: Alanine aminotransferase
ALB: Albumin
TBIL: Total bilirubin
ALP: Alkaline phosphatase
GGT: Gamma-glutamyl transferase
PT: Prothrombin time
INR: International normalized ratio
CREAT: Creatinine
BUN: Blood urea nitrogen
CPS: Child-Pugh score
RBC: Red blood cell
FFP: Fresh frozen plasma
Cryo: Cryoprecipitate
